# Dual Arch Rehabilitation With a Maxillary Cast Denture and a Mandibular Cast Partial Denture Incorporating Semi-precision Attachments: A Case Report

**DOI:** 10.7759/cureus.84990

**Published:** 2025-05-28

**Authors:** Amulya Jain, Aswini K Kar, Mousumi Mahato, Aditi Kulshrestha

**Affiliations:** 1 Prosthodontics, Kalinga Institute of Dental Sciences, Kalinga Institute of Industrial Technology (KIIT) Deemed to be University, Bhubaneswar, IND; 2 Prosthodontics, All India Institute of Medical Sciences, New Delhi, New Delhi, IND; 3 Dental Surgery, Srirama Chandra Bhanja (SCB) Dental College and Hospital, Cuttack, IND; 4 Endodontics, The Mini Tooth, Gurgaon, IND

**Keywords:** complete denture prosthesis, dental procedures, dental restoration, dental surgery, edentulous patients, geriatric dentistry, implantology dental education, patient care dental implants, patient management geriatric rehabilitation, prosthetics

## Abstract

The present case report depicts the prosthodontic treatment of a patient with an entirely edentulous maxilla and a Kennedy Class I partially edentulous mandible. The therapy comprised a maxillary cast complete denture and a mandibular cast partial denture (CPD) utilizing semi-precision attachments. The maxillary denture addressed the completely edentulous arch, and the semi-precision attachments in the mandibular CPD were used to enhance retention and distribute the load more effectively. The technique was intended to improve function, aesthetics, and stability to enhance the patient's quality of life.

## Introduction

Tooth loss in older adults has a significant bearing on oral function, phonetics, and quality of life, and thus demands complete prosthodontic rehabilitation. Complete edentulous maxillary and partial mandibular edentulous arch rehabilitation presents unique clinical challenges, particularly when anatomical limitations and altered morphology of the natural teeth are present. Kennedy Class I mandibular cases call for careful planning to achieve sufficient retention, support, and stability owing to the bilateral edentulous span and complete opposing edentulism [1].

Residual ridge resorption, especially in the chronically edentulous patient, complicates prosthetic planning and, in most cases, necessitates specialized prosthetic design or support measures [2]. In such cases, a detailed evaluation of residual ridge shape, mucosal health, and remaining dentition is warranted to create an effective and individualized prosthodontic plan. This case report presents the prosthodontic rehabilitation of a 65-year-old fully edentulous male patient with a fully edentulous maxilla and a Kennedy Class I partially edentulous mandible, with an emphasis on functional rehabilitation and clinical considerations for long-term stability.

## Case presentation

A 65-year-old male patient reported to the Department of Prosthodontics and Crown and Bridge, Kalinga Institute of Dental Sciences, Bhubaneswar, with the chief complaint of missing teeth, which led to impaired mastication, deglutition, and speech. On extraoral examination, the patient showed no facial asymmetry or temporomandibular joint (TMJ) dysfunction. Intraoral examination revealed that the maxillary arch was completely edentulous, presenting a well-rounded U-shaped form with firm and resilient oral mucosa. The mandibular arch was partially edentulous, corresponding to Kennedy's Class I classification, and exhibited a U-shaped but knife-edged residual ridge. The remaining anterior teeth (mandibular teeth 33-43) showed generalized attrition. The mucosa in both arches was firm and resilient. Radiographic evaluation revealed significant enamel loss in the mandibular anterior teeth, resulting in high pulpal horns. The patient was informed of all treatment options, including both fixed and removable prostheses. He opted for a removable prosthesis. A maxillary cast complete denture and a mandibular cast partial denture (CPD) with semi-precision attachments were selected as the treatment plan.

**Figure 1:**
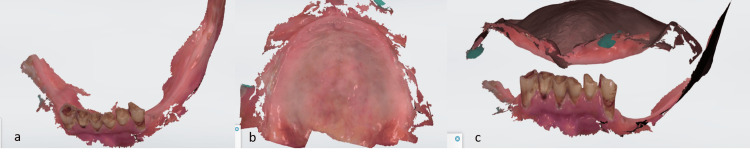
Intraoral scans of maxilla and mandible (3Shape Trios, Copenhagen, Denmark). (a) Preoperative mandibular intraoral scan. (b) Preoperative maxillary intraoral scan. (c) Preoperative interocclusal scan.

**Figure 2:**
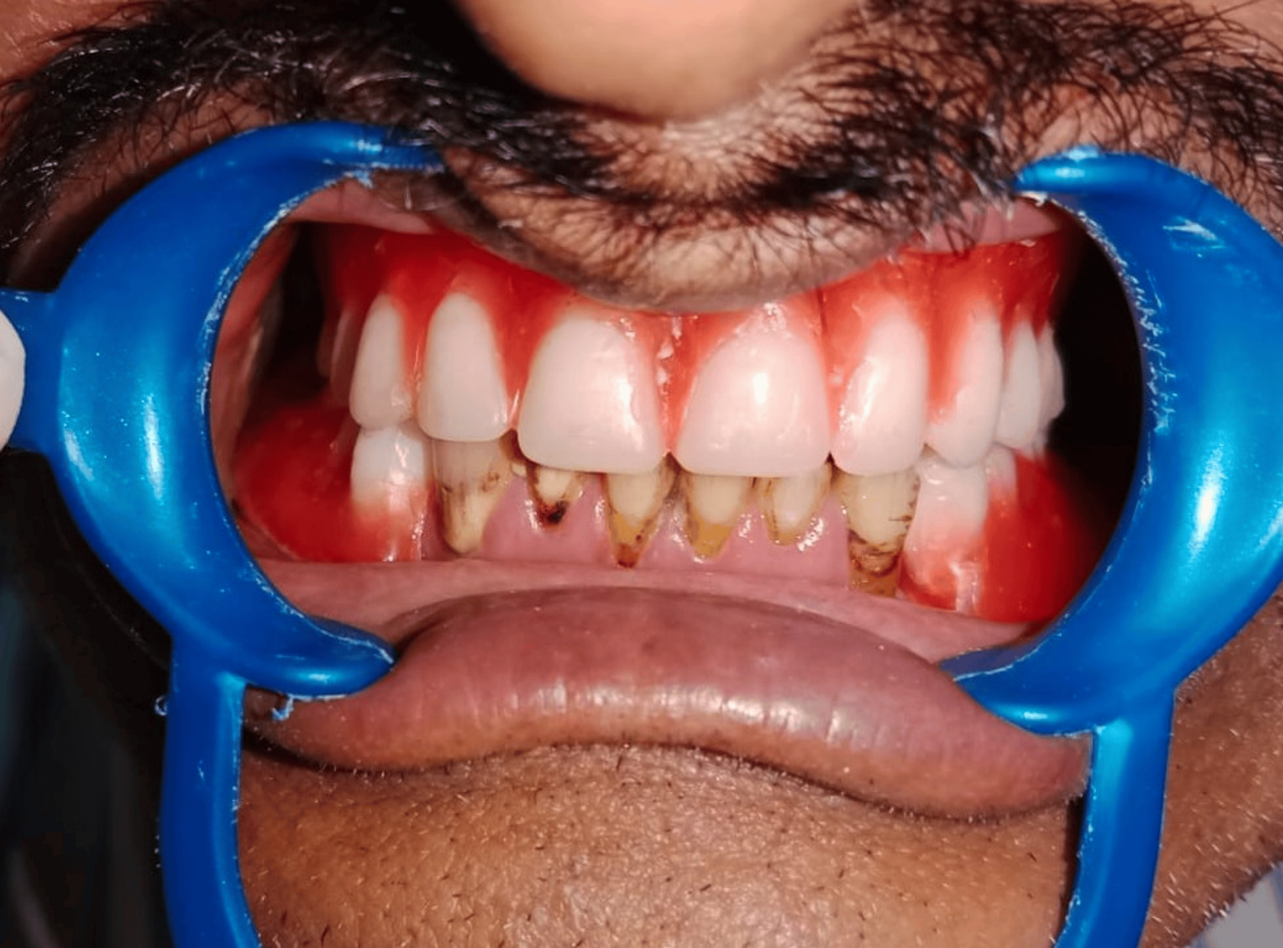
Arbitrary wax try-in.

Instead of traditional physical impressions, the maxilla and mandible were scanned using an intraoral scanner (3Shape Trios, Copenhagen, Denmark) (Figure 1). This digital method captured the precise shape and contours of the teeth and surrounding tissues. Based on the digital scan data (STL files), physical models (casts) of the patient's jaws were created using a 3D printer (Nestra 3D Printer). Jaw relation was recorded via the nick and notch method, and a facebow transfer (Hanau Wide Vue II) was performed to determine and record both the vertical dimension of occlusion and the centric relation or maximum intercuspation. The facebow transfer recorded the relationship of the patient's maxillary arch to the opening and closing axis of the TMJs. Before finalizing the design, especially for the fixed partial denture (the crowns on the lower front teeth in this case), an "arbitrary wax try-in" was performed (Figure 2). This allowed evaluation and adjustment of aspects like tooth position, shape, length, alignment, lip support, and overall aesthetics before fabricating the final restorations. It helped ensure that the planned treatment met both functional and aesthetic goals. Intentional root canal treatment (RCT) of the mandibular anterior teeth was performed, as significant alignment changes were needed. These changes required the removal of substantial tooth structure, potentially exposing or irritating the pulp. Prophylactic RCT was done to prevent future pain, infection, or the need for RCT after the crowns were cemented.

**Figure 3:**
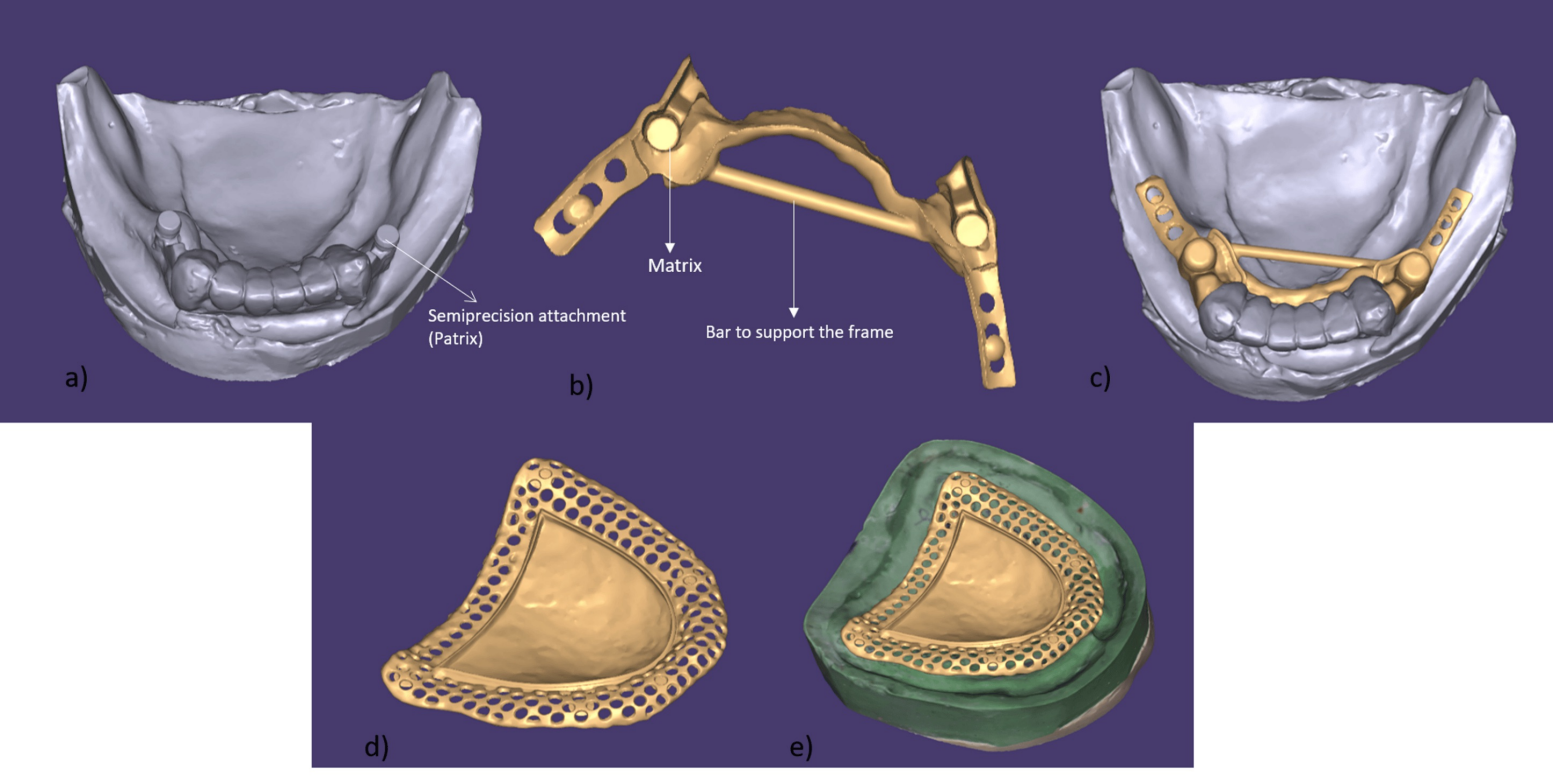
EXOCAD design of maxillary and mandibular prosthesis. (a) STL file of the mandibular arch. (b) Exocad design of the mandibular cast partial denture. (c) Exocad file showing the cast partial denture with the mandibular arch. (d) Exocad design of the maxillary cast denture. (e) Exocad file showing the cast denture with the maxillary arch. CAD: Computer-Aided Design; STL: Standard Triangle Language.

**Figure 4:**
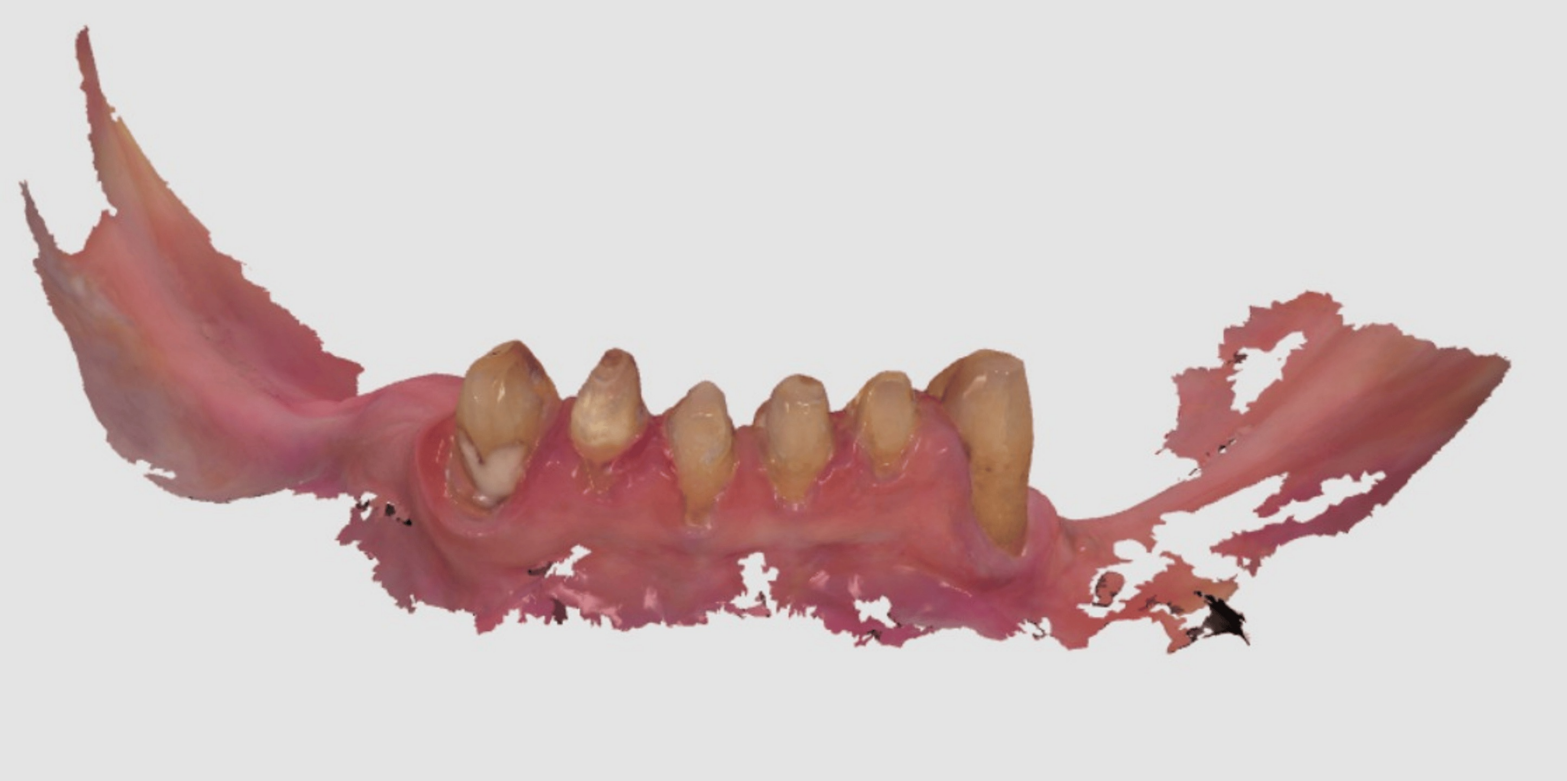
Intraoral scan of tooth preparation.

Semi-precision attachments are mechanical connectors consisting of two parts (e.g., a "male" part on the fixed crown and a "female" part within the removable partial denture). They were planned for the distal surfaces of the mandibular canines (teeth 33 and 43). These attachments provide retention and stability for the lower CPD. The prosthesis was designed using exocad software (exocad DentalCAD software 3.2 Elefsina) (Figure 3). A maxillary cast denture was designed, as the metal base offers advantages such as thinness (more tongue space, less bulk), better thermal conductivity, improved stability, durability, and potentially a better fit. A CPD was designed for the mandible to replace missing teeth. The major connector was a lingual bar, as it is rigid yet covers minimal tissue. The design also included minor connectors, the housings for the semi-precision attachments, and retention elements for the acrylic base and artificial teeth. The mandibular anterior teeth (33-43) were prepared for porcelain-fused-to-metal (PFM) crowns, ensuring the crowns had adequate retention and resistance to dislodging forces (Figure 4).

**Figure 5:**
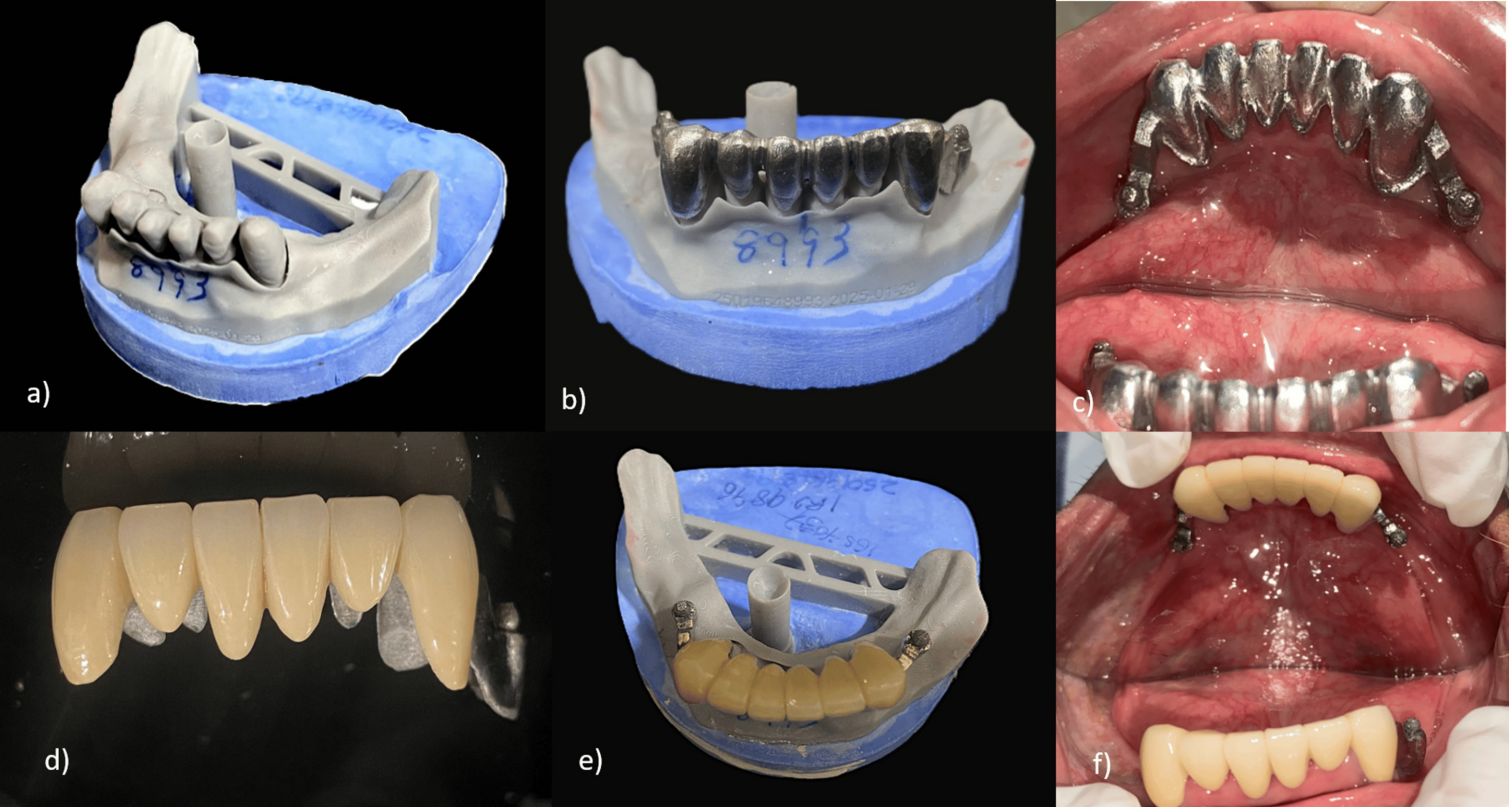
Cementation of PFM crowns. (a) 3D-printed cast after intraoral scanning of tooth preparations 43-33. (b) Metal framework of the fixed prosthesis. (c) Intraoral metal try-in of the crowns. (d) Fabricated crowns with respect to 33-43. (e) Fabricated crowns on the 3D-printed cast. (f) Final cementation of the crowns. PFM: Porcelain-Fused-to-Metal.

**Figure 6:**
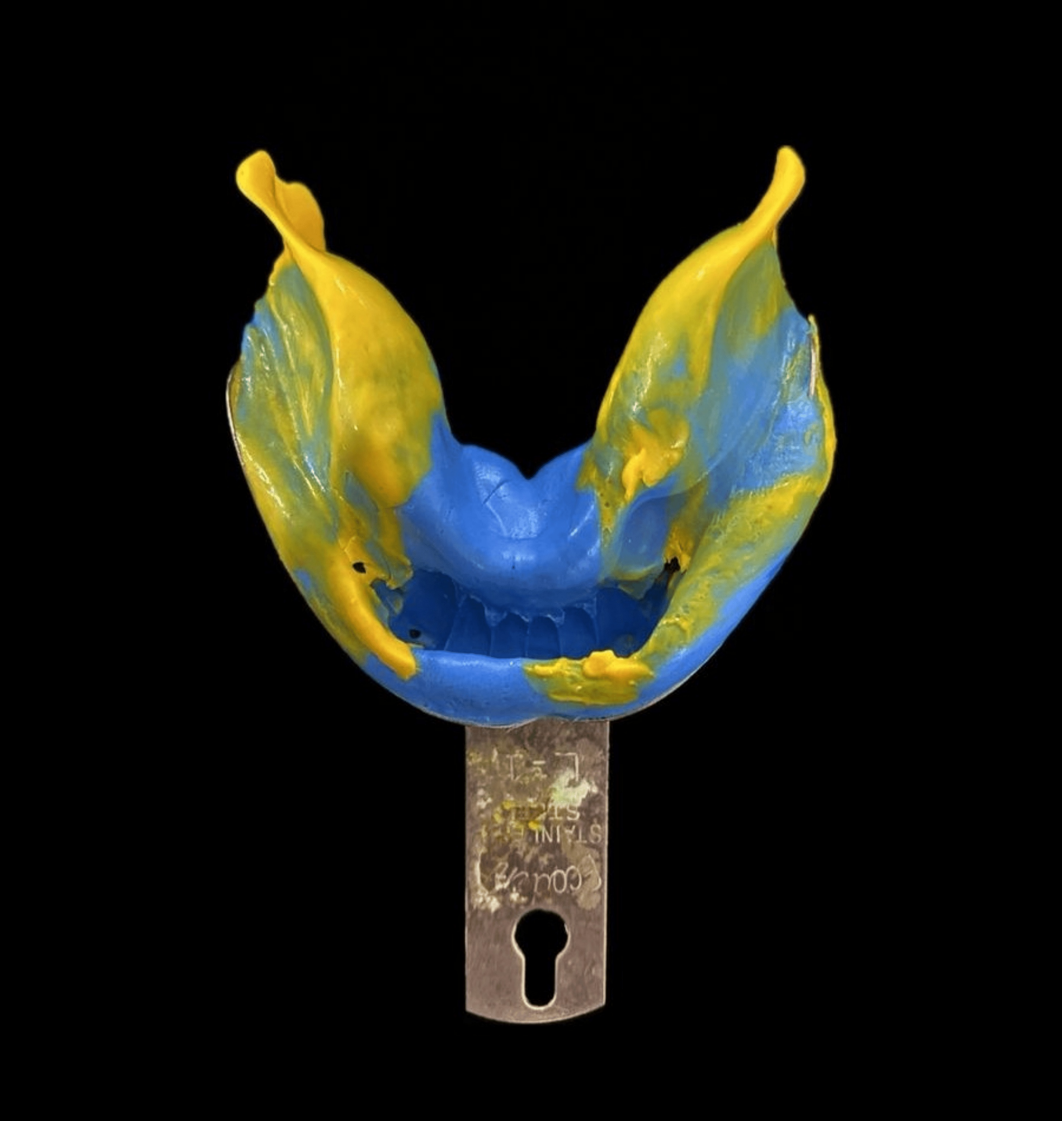
Final elastomeric impression.

**Figure 7:**
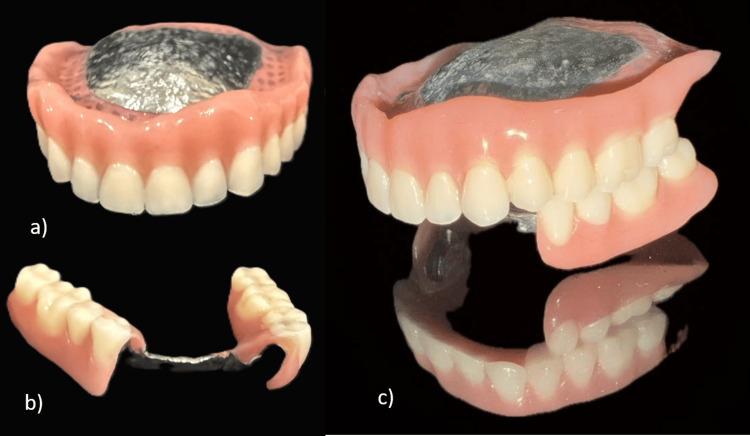
Fabrication of prosthesis. (a) Final maxillary prosthesis. (b) Final mandibular prosthesis. (c) Maxillary and mandibular prostheses in occlusion.

The PFM crowns with the patrix of the semi-precision attachments on the distal surfaces of 33 and 43 were cemented using glass ionomer cement onto the prepared lower anterior teeth (Figure 5). This step established the final position and contour of the abutment teeth that would support the partial denture. After the crowns with their attachments were cemented, a final, highly accurate impression of the entire mandibular arch was taken using an elastomeric impression material (Waldent Flexident A-Silicone Putty) (Figure 6). This impression ensured that the CPD would fit precisely onto the supporting structures and engage correctly with the attachments. A full-arch, highly accurate elastomeric impression was made after the abutment crowns with semi-precision attachments were cemented. This approach captured the final relationship of the abutments and soft tissues in a single definitive impression, rather than using the two-stage impression process characteristic of the altered cast technique. The altered cast technique was not used, as it involves fabricating the metal framework first on an initial cast. Then, the framework is used with custom trays attached to its distal extensions to make a secondary impression of the edentulous ridges, often under some degree of functional loading. The original cast is then "altered" with this new ridge information. Using the final impressions, jaw relation records, facebow transfer data, and digital designs, the cast metal maxillary denture and mandibular CPD were fabricated, and acrylic resin and artificial teeth were processed onto them (Figure 7). Bilateral balanced occlusion was chosen to provide simultaneous anterior and posterior contacts in centric as well as eccentric positions. This design helps distribute occlusal loads evenly, reduces tipping forces against the complete denture, and enhances retention. There was anterior guidance by the mandibular anterior natural teeth, which was well coordinated with the posterior occlusion to avoid maxillary denture displacement during lateral and protrusive movements. Cuspal anatomy and occlusal morphology were adjusted during the try-in and delivery steps to ensure even contact and minimize lateral stresses. In addition, the use of semi-precision attachments added stability during function, allowing the mandibular prosthesis to co-exist with the maxillary denture.

**Figure 8:**
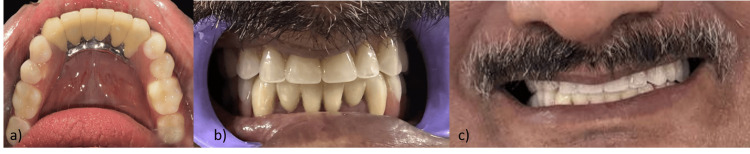
Insertion of prosthesis. (a) Intraoral occlusal view of the mandibular prosthesis. (b) Intraoral frontal view of the maxillary and mandibular prostheses. (c) Postoperative picture of the patient.

The completed maxillary cast denture and mandibular CPD were delivered to the patient. The fit, stability, and retention of both appliances were checked, and adjustments were made to ensure proper occlusion, comfort, and aesthetics (Figure 8). The function of the semi-precision attachments was verified. The patient was provided with instructions on how to insert, remove, and maintain the new prostheses.

## Discussion

In the current clinical case, the patient presented with a completely edentulous maxillary arch and a mandibular Kennedy Class I partially edentulous arch, retaining anterior teeth (33-43) with generalized attrition. A variety of treatment alternatives were evaluated, each with specific clinical implications. One such option was a conventional clasp-retained RPD, which is cost-effective and has straightforward fabrication. However, its disadvantages include poor esthetics due to visible clasps, reduced retention and stability in Kennedy Class I cases, and increased torque on abutment teeth. Given the patient’s desire for improved function and esthetics, this option was deemed suboptimal. Another treatment option was implant-supported overdentures (for the mandible and/or maxilla), which significantly improve retention, stability, and masticatory efficiency; reduce ridge resorption; and result in higher patient satisfaction. However, drawbacks include the invasive nature of the surgical procedure, higher cost, longer treatment time, and the need for sufficient bone volume. An implant-supported fixed prosthesis was also considered. It provides excellent function, esthetics, and long-term durability and does not require removal. However, it is the most invasive and expensive option, requiring multiple implants and posing challenges in hygiene maintenance, especially in elderly patients. Although ideal biomechanically, the patient declined surgical options and expressed a preference for removable solutions, rendering this approach inappropriate. A telescopic crown-supported removable prosthesis was another alternative, offering superior retention and load distribution. Telescopic crowns allow passive insertion and good hygiene access, but the option is technically demanding, expensive, and time-consuming.

The treatment plan decided upon involved the construction of a maxillary complete denture and a mandibular CPD with semi-precision attachments. Since the maxilla was completely edentulous with a well-rounded, U-shaped residual ridge and firm, resilient mucosa, a cast complete denture was considered a predictable, cost-effective solution for restoring function and esthetics [1]. The presence of favorable ridge anatomy contributes to better retention, support, and stability of the prosthesis [2]. Kennedy Class I in the mandibular arch presents certain biomechanical challenges because of the bilateral distal extension and lack of posterior abutments, which can result in instability and movement of the prosthesis during function [3]. In such cases, controlling prosthesis movement is essential. Semi-precision attachments assist in stabilizing the prosthesis against rotational and vertical forces [4,5]. They enhance retention, esthetics, and load distribution through mechanical interlocking between the prosthesis and abutments, independent of visible clasps [4,6]. Semi-precision attachments also reduce torque on abutment teeth and help sustain a favorable stress pattern throughout the arch, which is essential where supporting tissues are deficient, such as in a knife-edge mandibular ridge [6,7]. Preservation of anterior teeth aids in ridge preservation, proprioception, and esthetic integration, while providing vertical support and indirect retention to the CPD [3,8]. In Kennedy Class I cases, effective regulation of prosthesis movement is vital. Semi-precision attachments help retain the prosthesis against rotation and vertical displacement [4,7]. This treatment plan also permits bilateral balanced occlusion, which is important for function and comfort, particularly when coordinated with a maxillary complete denture [2].

Recent systematic reviews indicate a growing trend toward the use of semi-precision attachments in removable partial dentures (RPDs) due to their esthetic and biomechanical benefits. Awawdeh M et al. [9] concluded that attachment-retained RPDs demonstrate greater patient satisfaction and reduced maintenance needs compared to clasp-retained dentures. These attachments distribute occlusal forces more evenly, minimize torque on the abutment teeth, and provide better esthetics by eliminating visible clasps [10]. Kennedy Class I mandibular cases pose a unique biomechanical challenge due to distal extension and the absence of posterior abutments. Semi-precision attachments have been shown to effectively reduce rotational movement and improve masticatory efficiency in Kennedy Class I cases compared to RPI clasp designs and stress-breaker systems [11]. Digital dentistry has transformed prosthodontic treatment planning and delivery. Intraoral scans and CAD/CAM fabrication methods continue to improve fit accuracy and reduce chair time. Patient satisfaction is one of the major indicators of prosthodontic success [12]. Patients with attachment-retained RPDs have reported higher satisfaction scores in areas such as comfort, chewing function, speech, and esthetics [11].

The long-term success of the chosen prosthetic treatment is favorable, provided that proper maintenance and recall schedules are followed [13]. While semi-precision attachments are highly durable, they may show signs of wear over time, especially in patients with high occlusal forces or parafunctional habits. The most frequently encountered complications include loss of retention due to wear of the attachment matrix, breakage of the acrylic base, and loosening of abutment crowns. To prevent these complications, periodic recall appointments should be planned, and worn plastic or nylon matrix components should be replaced as needed. The patient was instructed on how to clean under and around the attachments. Periodic radiographic examinations are recommended to evaluate periodontal health and the integrity of root canal-treated teeth. In the event of complications such as wear or fracture of attachments, chairside repair and component replacement are possible without the need to remanufacture the entire prosthesis. Overall, when well maintained, semi-precision attachment-retained prostheses offer a stable, esthetic, and functionally reliable long-term solution.

## Conclusions

This case illustrates a clinically effective method of rehabilitating a Kennedy Class I mandibular arch that is opposing a fully edentulous maxilla through the use of a CPD with semi-precision attachments and a maxillary complete cast denture. Although the treatment modality selected seemed to restore function and esthetics successfully in this one patient, these results are predicated only on descriptive observation with no objective measures or longitudinal data. Given the inherent limitations of a case report format, such as the absence of standardized outcome measures and lack of quantitative analysis, conclusions should be interpreted with caution. While the approach described may serve as a useful clinical guide, further studies involving systematic analysis, larger sample sizes, and measurable outcomes are necessary to establish the clinical efficacy and reproducibility of this treatment technique.
